# Editorial: Metabolic associated fatty liver disease (MAFLD) in childhood: a new challenge

**DOI:** 10.3389/fendo.2025.1569972

**Published:** 2025-02-19

**Authors:** Anna Di Sessa

**Affiliations:** Department of Woman, Child, and General and Specialized Surgery, University of Campania “Luigi Vanvitelli”, Naples, Italy

**Keywords:** MAFLD (metabolic associated fatty liver disease), metabolic dysfunction, children, complications, risk factors, epidemiology, early diagnosis, management

The recent nomenclature change from Non-Alcoholic Fatty Liver Disease (NAFLD) to Metabolic Associated Fatty Liver Disease (MAFLD) (1), and more recently to Metabolic Dysfunction-Associated Steatotic Liver Disease (MASLD) (2), has highlighted the growing medical significance of this condition, particularly in childhood, due to its substantial cardiometabolic burden (3). Indeed, the shift in terminology emphasizes the metabolic dysfunction underlying the condition, which is crucial for improving diagnosis and treatment (1, 2). Pediatricians face unique challenges in addressing this disease, underscoring the need for a deeper understanding to improve management strategies and patient outcomes (3).

Recent epidemiological data from Lin et al. examined the global prevalence of NAFLD and Non-Alcoholic SteatoHepatitis (NASH) in adolescents, based on body mass index (BMI), from 1975 to 2016, with projections through 2030. The global NAFLD prevalence was found to be 15.31% in boys and 12.68% in girls, while NASH prevalence was 2.50% in boys and 2.47% in girls. Both conditions increased with higher BMI and age, with higher rates observed in high-income Western countries, and lower rates in South Asia and Sub-Saharan Africa. The study predicts a continued rise in their prevalence through 2030, with BMI being a key determinant.

Consistent with these findings, a large Finnish retrospective study by Riekki et al. on 703 overweight children aged 2-16 years found an overall prevalence of MAFLD of 15%, with higher rates in boys (18%) than in girls (11%). MAFLD prevalence varied by puberty and gender, peaking in girls during early puberty and increasing in boys with age and puberty. Key risk factors included type 2 diabetes (T2D), impairments in glucose and lipid metabolism, and elevated BMI, with gender-specific patterns. In boys, MAFLD was strongly associated with postpubertal stage, elevated serum insulin, hypertriglyceridemia, and low HDL-cholesterol (HDL-c), while in girls, T2D, hypertriglyceridemia, and low HDL were the most significant factors.

Given the importance of early diagnosis, identifying useful markers is crucial (3). Weng et al. examined the relationship between arm circumference (AC) and NAFLD in a cohort of 1,559 American children and adolescents using data from the 2017–2020 NHANES survey. The study found a positive correlation between AC and the risk of NAFLD, with AC also linked to more severe liver steatosis. In addition, authors identified a non-linear relationship, noting an inflection point at 34.5 cm. Due to its independent association with elevated NAFLD risk, AC could serve as a valuable marker for assessing the disease in childhood.

Moreover, the use of bioelectrical impedance analysis (BIA) for screening MASLD in overweight and obese youth has been also examined in a study of Song et al. An analysis of 206 children and adolescents found that parameters such as waist-to-hip ratio (WHR), body fat percentage (PBF), and BIA measurements were significantly correlated with elevated ALT levels and MASLD scores. Notably, the study identified strong associations between WHR, PBF-WHR, and visceral fat area/WHR (VFA-WHR) with MASLD risk, even in individuals with normal ALT levels. These findings suggest that BIA-based screening, when combined with anthropometric measurements, could serve as a valuable, non-invasive tool for identifying youth at risk for MASLD, enabling earlier detection and intervention.

Interestingly, Wang et al. explored the relationship between MAFLD and sex hormones, particularly sex hormone-binding globulin (SHBG) in 155 male children with obesity clustered in two groups as “MAFLD” and “simple obesity” Boys with MAFLD exhibited higher BMI, insulin levels, and liver damage markers such as transaminases, but lower levels of HDL-c, testosterone, and SHBG compared to those with simple obesity. Notably, BMI, testosterone, and SHBG were identified as independent risk factors for MAFLD, highlighting their potential utility in early detection and targeted interventions.

In the wide cardiometabolic spectrum of the disease, Polycystic ovary syndrome (PCOS) is often linked to MAFLD, affecting both liver function and metabolic processes (3).

An insightful study by Garcia-Beltran et al. investigated the effects of oral contraceptive (OC) treatment versus a spironolactone-pioglitazone-metformin combination (spiomet) on organokines in adolescent girls with PCOS but without obesity as well as their relationship with liver damage biomarkers. After 6 months, OC treatment resulted in increased levels of alanine aminotransferase (ALT) and gamma-glutamyl transferase (GGT), whereas spiomet did not affect these markers. Fibroblast growth factor-21 (FGF21) levels were higher in girls with PCOS, while diazepam-binding protein-1 (DBI) levels were lower in those treated with OC compared to controls. The increase in ALT and GGT in the OC-treated group was associated with elevated meteorin-like protein (METRNL) levels, suggesting that METRNL may play a role in the hepatic changes induced by OC treatment.

Noteworthy, MAFLD may also complicate severe systemic diseases. Hepatopulmonary syndrome (HPS) is increasingly recognized as a rare but serious MAFLD complication even in childhood. Choe et al. described a case of HPS in an 18-year-old female with MAFLD following craniopharyngioma resection and hypopituitarism. The patient was successfully treated with growth hormone replacement therapy (GHRT), showing significant improvement in respiratory symptoms and normalization of lung shunt ratio after 6 months, without the need for liver transplantation. A systematic review of 9 additional pediatric cases of HPS linked to hypopituitarism found that GHRT absence may have contributed to HPS development. Although three patients underwent liver transplantation, all had a recurrence of NASH. Therefore, GHRT could be an effective alternative to liver transplantation in treating HPS, but its efficacy needs to be further confirmed.

Consistent with these findings, Yoshikawa et al. reported a case of a 13-year-old Asian boy with hypothalamic obesity secondary to panhypopituitarism resulting from craniopharyngioma, who developed HPS. Despite normal growth despite growth hormone (GH) deficiency, GH replacement therapy was not initially considered. However, the patient developed HPS despite successfully managing his body weight, suggesting that weight reduction alone—typically the first-line treatment for MAFLD—may not suffice, and GH replacement therapy could be necessary for improved outcomes.

In light of its emerging role in overall cardiometabolic health from childhood onward (3), this Research Topic aims to highlight the complexity of MAFLD as an intriguing multisystem disease.
